# A study of pressure-driven flow in a vertical duct near two current-carrying wires using finite volume technique

**DOI:** 10.1038/s41598-022-25756-4

**Published:** 2022-12-08

**Authors:** Kashif Ali, Wasim Jamshed, S. Suriya Uma Devi, Rabha W. Ibrahim, Sohail Ahmad, El Sayed M. Tag El Din

**Affiliations:** 1 Department of Basic Sciences and Humanities, Muhammad Nawaz Sharif University of Engineering and Technology, Multan, 60000 Pakistan; 2 Department of Mathematics, Capital University of Science and Technology (CUST), Islamabad, 44000 Pakistan; 3 Department of Mathematics, KPR Institute of Engineering and Technology, Coimbatore, 641407 India; 4 Water Resources and Applied Mathematics Research Lab, Nagpur, 440027 India; 5 Centre for Advanced Studies in Pure and Applied Mathematics, Bahauddin Zakariya University, Multan, Pakistan; 6 Electrical Engineering, Faculty of Engineering and Technology, Future University in Egypt, New Cairo, 11835 Egypt

**Keywords:** Mathematics and computing, Physics

## Abstract

For heating, ventilation or air conditioning purposes in massive multistory building constructions, ducts are a common choice for air supply, return, or exhaust. Rapid population expansion, particularly in industrially concentrated areas, has given rise to a tradition of erecting high-rise buildings in which contaminated air is removed by making use of vertical ducts. For satisfying the enormous energy requirements of such structures, high voltage wires are used which are typically positioned near the ventilation ducts. This leads to a consequent motivation of studying the interaction of magnetic field (MF) around such wires with the flow in a duct, caused by vacuum pump or exhaust fan etc. Therefore, the objective of this work is to better understand how the established (thermally and hydrodynamically) movement in a perpendicular square duct interacts with the MF formed by neighboring current-carrying wires. A constant pressure gradient drives the flow under the condition of uniform heat flux across the unit axial length, with a fixed temperature on the duct periphery. After incorporating the flow assumptions and dimensionless variables, the governing equations are numerically solved by incorporating a finite volume approach. As an exclusive finding of the study, we have noted that MF caused by the wires tends to balance the flow reversal due to high Raleigh number. The MF, in this sense, acts as a balancing agent for the buoyancy effects, in the laminar flow regime

## Introduction

When a flow is fully developed (both hydrodynamically and thermally), it is said to have reached steady state. For the given constant heat flux, there will be no temperature variation with respect to time. Gevari et al.^1^ presented a review paper in this direction describing the direct and indirect thermal applications of hydrodynamic and acoustic cavitation. Menni et al.^2^ introduced an analysis of the hydrodynamic and thermal of water, ethylene glycol and water-ethylene glycol as base liquids isolated by aluminum oxide nano-measured dense particles. Tayeb et al.^3^ evaluated the hydrodynamic and thermal performances of nanofluids (NFs) in a chaotic situation. Phan et al.^4^ prepared a numerical investigation on concurrent thermodynamic and hydrodynamic instruments of subaquatic explosion. Another presentation of numerical studied on heat transfer (HT) performance of thermally mounting movement inside rectangular microchannels is given by Ma et al.^5^. Mozaffari et al.^6^ increased the ability of lattice using the mechanism of hydrodynamic and thermal. Wakif et al.^7^ checked the stability of thermal radiation and surface roughness effects via the thermo-magneto-hydrodynamic method. Ali et al.^8^ formulated a new mathematical revision of MF communication with completely established movement in a vertical duct. Rios et al.^9^ formulated an investigational assessment of the current and hydrodynamic presentation of NFs in a coiled flow inverter. Sabet et al.^10^ studied the behavior of the current and hydrodynamic of forced convection steamy slide flow in a metal spray.

Current-carrying wire model (CCWM) is used in fluid for many advantages. Its usability appeared in many researches, where a review paper in this direction is given by Liu et al.^11^. A case study is introduced for shape memory of NFs by Osorio et al.^12^ and Zareie, et al.^13^. Azmi et al.^14^ studied HT for hybrid nanofluids (HNFs) in a tube with CCWM. Kumar and Sharma^15^ optimized ferrofluid using CCWM. Numerical and computational investigations are presented using the advantages of CCWM by Khan et al.^16^ on a constant fluid, Ali et al.^8^ for MF interaction, Chang et al.^17^ on magnetic NFs. He et al.^18^ introduced a computational heat transfer and fluid flow in view of CCWM. Lu et al.^19^ gave a computational fluid dynamics examination of a dust scrubber with CCWM. Briggs and Mestel^20^ showed a linear stability of a ferrofluid centered on a CCWM. Dahmaniet al.^21^ enhanced the HT of ferrofluid movement in a solar absorber tube by a periodic CCWM. Sharma et al.^22^ analyzed the MF-strength of multiple coiled utilizing the idea of CCWM. Vinogradova et al.^23^ modeled a system of ferrofluid-based microvalves in the MF shaped by a CCWM. He et al.^24^ studied the dynamic pull-in for micro–electromechanical scheme with a CCWM.

A magnetic field (MF), which can be thought of as a vector field, governs the magnetic effect on stirring rechargeable tasks, power-driven flows, and magnetic resources. An influencing control in an MF involves a force that is perpendicular to both the control's own velocity and the MF. Zhang et al.^25^ examined the HNFs movement near an adaptable insincere with tantalum and nickel NFs, according to the consequence of MF. Talebi et al.^26^ offered an inspection of mixture-based opaque HNF movement in porous mass media inflated by MF operating mathematical technique. Ayub et al.^27^ deliberated the MF of nanoscale HT of magnetized 3-D chemically radiative HNF. Mourad et al.^28^ employed the finite element analysis of HT of Fe3O4-MWCNT/water HNF engaged in curved addition with uniform MF. Manna et al.^29^ showed a novel multi-banding application of MF to convective transport arrangement employed with porous medium and HNF. Khashi’ie et al.^30^ examined unsteady hugging movement of Cu-Al2O3/water HNF in a straight channel with MF. Lv et al.^31^, Khan et al.^32^ and Alkasasbeh et al.^33^ distributed a numerical technique near microorganisms HNF movement with the arcade current and MF over a revolving flappy. Roy et al.^34^ investigated HT of MHD dusty HNFs over a decreasing slide. Khazayinejad and Nourazar^35^ recycled the fractional calculus to describe 2D-fractional HT examination of HNF alongside a leaky plate together with MF. Gürdal et al.^36^ compressed the HNF curving in depressed tube imperiled with the MF. Azad et al.^37^ presented a study on rapid and sensitive MF sensor based on photonic crystal fiber with magnetic fluid infiltrated nanoholes. Skumiel et al.^38^ considered the consequence of the MF on the thermal effect in magnetic fluid. Alam et al.^39^ examined the influence of adjustable MF on viscous fluid between 3-D rotatory perpendicular hugging platters.

In multistory, enormous building constructions, vertical duct geometry (VDG) is channels or paths utilized to deliver, reappearance, or use air for reheating, ventilation, or air conditioning. Rapid population expansion, particularly in areas with concentrated industries, has given rise to a culture of building skyscrapers with tens of stories, where vertical ducts are the obvious select for eliminating muted air. Ranjbar et al.^40^ enhanced the wind turbine equipped with a VDG. Kim et al.^41^ presented a computational fluid dynamics analysis of buoyancy-aided turbulent mixed convection inside a heated VDG. López et al.^42^ designed selection and geometry in OWC wave dynamism converters for performance. Umavathi and Bég^43^ introduced a computation of thermo-solutal convection with soret-dufour cross diffusion in a VDG NFs. Oluwade and Glakpe^44^ computed 3D-Mixed convection in a VDG. Choudhary^45^ optimized the VDG of 3D printer part cooling fan duct. Li et al.^46^ studied the effects of VDG on intraglottal pressures in the convergent glottis. Zhao et al.^47^ investigated of necessary instrument leading to the performance development of VDG. Wojewodka et al.^48^ considered a numerical study of complex flow physics and coherent structures of the flow through a longwinded VDG. Moayedi and Amanifard^49^ enhanced the electrohydrodynamic usual HT in a VDG.

A technique for expressing and analyzing partial differential equations as algebraic equations is known as the finite volume method (FVM)^50^. The divergence theorem is used in the finite volume method to transform volume integrals in a partial differential equation containing a divergence term into surface integrals. The surfaces of each finite volume are then used to evaluate these terms as fluxes. Namdari et al.^51^ investigated of the effect of the discontinuity direction on fluid flow in porous rock masses on a large-scale using HNFs and streamline utilizing FVM. Faroux et al.^52^ studied a coupling non-local rheology and capacity of liquid (VOF) process in view of FVM implementation. Xu et al.^53^ simulated a system of incompressible curved element hydrodynamics‐finite volume technique joining procedure for interface tracking of two‐phase fluid movements in view of FVM. Wang et al.^54^ investigated a coupled optical-thermal-fluid-mechanical analysis of parabolic trough solar receivers employing supercritical CO_2_ as HT in virtue of FVM. Liu et al.^55^ studied the consequence of gas compressibility on liquid ground of air‐cooled turbo‐generator according to FVM. Koulali et al.^56^ presented a comparative study on effects of thermal gradient direction on heat exchange between a pure fluid and NFs hiring FVM. Ding et al.^57^ considered a mathematical examination of passive toroidal tuned liquid column dampers for the trembling regulator of monopile wind turbines using FVM and FEM. Makauskas^58^ indicated a comparison of FDM, FVM with NN for solving the forward problem. Yousefzadeh et al.^59^ inspected a natural convection of Water/MWCNT NF movement in an inclusion for examination of the first and second laws of thermodynamics in view of FVM.

In this work, the complex interaction of thermodynamically as well as hydrodynamically settled current in a perpendicular square channel, with the MF created by neighboring positioned two wires, has been investigated for the first time. One wire is positioned whereas the other one is assumed to be present above the duct. The new aspects of the issue are described through physical explanations. A finite volume based computational approach has been developed to obtain the numerical solution for different values of the governing parameters. The numerical results have been depicted in the graphical form, and are interpreted accordingly.

## Problem formulation

**Figure 1:**
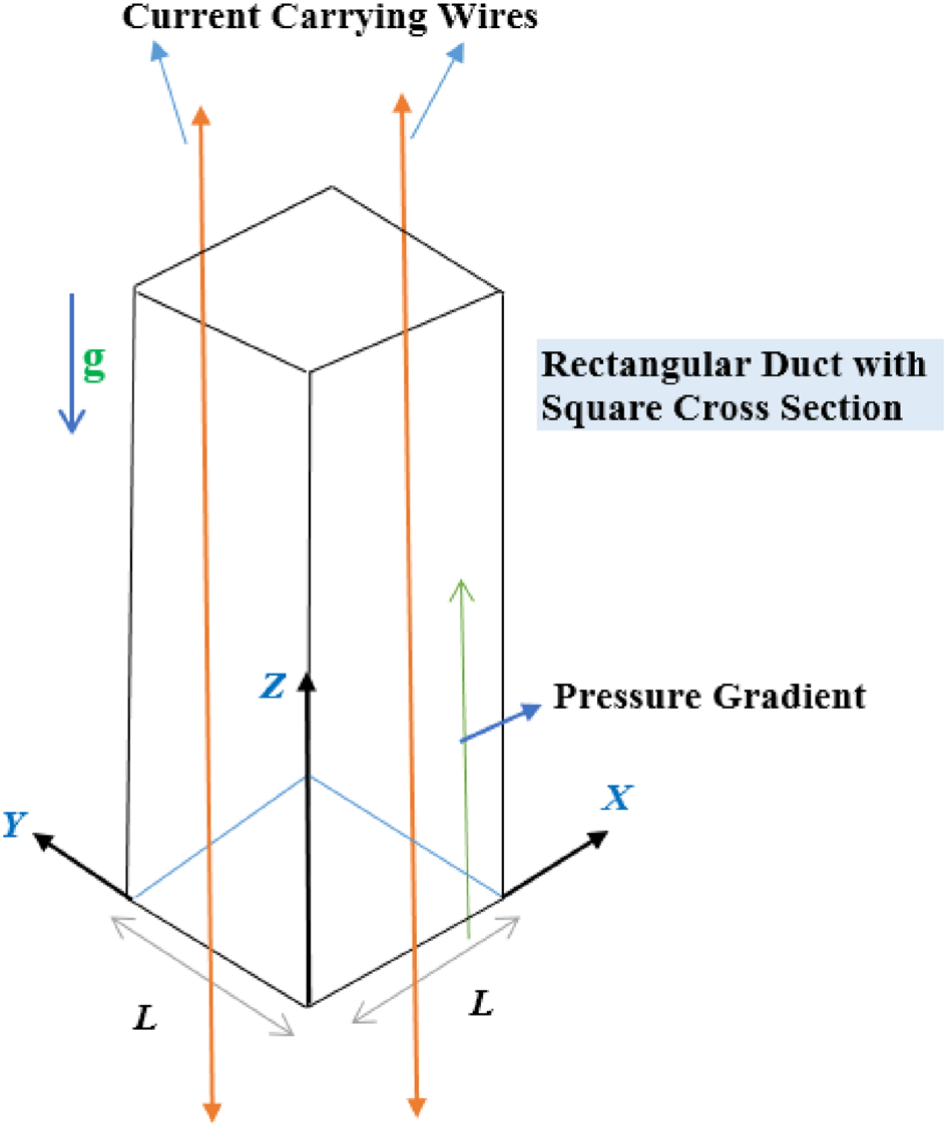
Physical model of the problem.

We consider the fully settled movement of a standard Newtonian fluid (in a perpendicular square channel with side *L*) based on the exterior pressure gradient. The current is expected to be stable, laminar and incompressible. That is why, the velocity is:

1 $$\vec{V} = \left( {w_{1} ,\,w_{2} ,\,w_{3} } \right) = \left( {0,\,0,\,w_{3} \left( {y_{1} ,\,y_{2} } \right)} \right),$$

with $y_{1} ,\,y_{2} \,{\text{and}}\,y_{3}$ being the standard three co-ordinate directions. Liquid possessions specifically, the current diffusivity, the thermal conductivity, and the dynamic viscosity are supposed to be non-varying. The necessary (geometrical) distorted example is exposed as in Fig. 1.

Usually, the buoyancy-driven flows involve the idea of Boussinesq approximation. This approximation reveals the relation between inertia and gravity. In Boussinesq hypothesis, gravity is considered to be large but inertia is negligibly small. With viscous dissipation being deserted and the Boussinesq suggestion being applied, the accurate design of the problematic comprising of Navier-Stokes equations and the energy stability equation are^8,60^:

2 $$\frac{{\partial u_{1} }}{{\partial y_{1} }} + \frac{{\partial u_{2} }}{{\partial y_{2} }} + \frac{{\partial u_{3} }}{{\partial y_{3} }} = 0,$$

3 $$\rho \left( {u_{1} \frac{{\partial u_{1} }}{{\partial y_{1} }} + u_{2} \frac{{\partial u_{2} }}{{\partial y_{2} }} + u_{3} \frac{{\partial u_{3} }}{{\partial y_{3} }}} \right) = - \frac{\partial p}{{\partial y_{1} }} + \mu \left( {\frac{{\partial^{2} u_{1} }}{{\partial y_{1}^{2} }} + \frac{{\partial^{2} u_{1} }}{{\partial y_{2}^{2} }} + \frac{{\partial^{2} u_{1} }}{{\partial y_{3}^{2} }}} \right),$$

4 $$\rho \left( {u_{1} \frac{{\partial u_{2} }}{{\partial y_{1} }} + u_{2} \frac{{\partial u_{2} }}{{\partial y_{2} }} + u_{3} \frac{{\partial u_{2} }}{{\partial y_{3} }}} \right) = - \frac{\partial p}{{\partial y_{2} }} + \mu \left( {\frac{{\partial^{2} u_{2} }}{{\partial y_{1}^{2} }} + \frac{{\partial^{2} u_{2} }}{{\partial y_{2}^{2} }} + \frac{{\partial^{2} u_{2} }}{{\partial y_{3}^{2} }}} \right),$$

5 $$\rho \left( {u_{1} \frac{{\partial u_{3} }}{{\partial y_{1} }} + u_{2} \frac{{\partial u_{3} }}{{\partial y_{2} }} + u_{3} \frac{{\partial u_{3} }}{{\partial y_{3} }}} \right) = - \frac{\partial p}{{\partial y_{3} }} + \mu \left( {\frac{{\partial^{2} u_{3} }}{{\partial y_{1}^{2} }} + \frac{{\partial^{2} u_{3} }}{{\partial y_{2}^{2} }} + \frac{{\partial^{2} u_{3} }}{{\partial y_{3}^{2} }}} \right) + \left( {\rho \beta } \right)g\left( {T - T_{0} } \right) - \sigma \overline{B}^{2} u_{3} ,$$

6 $$\left( {u_{1} \frac{\partial T}{{\partial y_{1} }} + u_{2} \frac{\partial T}{{\partial y_{2} }} + u_{3} \frac{\partial T}{{\partial y_{3} }}} \right) = \alpha \left( {\frac{{\partial^{2} T}}{{\partial y_{1}^{2} }} + \frac{{\partial^{2} T}}{{\partial y_{2}^{2} }} + \frac{{\partial^{2} T}}{{\partial y_{3}^{2} }}} \right),$$

where the flow pressure, heat. Current diffusivity and the density of the fluid are signified by their familiar normal signs. The Eq. (2) represents the equation of continuity or mass conservation equation. The constant of a pyro-magnetic coefficient is denoted by β which measures the magnetization for different temperature curves. $\sigma$ is the electrical diffusivity of the NF, and $\overline{B} = \overline{\mu }_{0} \overline{H}$ is the MF induction with $\overline{H}$ being the MF intensity due to the current carrying wires positioned at $\left( {y_{1}^{1} ,\,y_{2}^{1} } \right)$ and $\left( {y_{1}^{2} ,\,y_{2}^{2} } \right)$ with

$$\overline{H} = \sqrt {\overline{H}_{1}^{2} + \overline{H}_{2}^{2} }$$ where

$$\overline{H}_{1} = \frac{\gamma }{2\pi }\frac{1}{{\sqrt {\left( {y_{1} - y_{1}^{0} } \right)^{2} + \left( {y_{2} - y_{2}^{0} } \right)^{2} } }},\,\overline{H}_{2} = \frac{\gamma }{2\pi }\frac{1}{{\sqrt {\left( {y_{1} - y_{1}^{1} } \right)^{2} + \left( {y_{2} - y_{2}^{1} } \right)^{2} } }}$$

Further, $T_{0}$ characterizes an orientation heat which is selected in such a way that there exists a linear association between the local heat and the local mass density. The term $\gamma$ represents the magnetic field strength associated with external current. A typical select for the orientation heat is:

7 $$T_{0} = \frac{1}{{L^{2} }}\int\limits_{0}^{L} {\int\limits_{0}^{L} {T\,dy_{1} \,dy_{2} ,} }$$

This is the mean movement heat in the channel at a specific fractious section. The proposed movement field and the choice $$\left( {{\raise0.7ex\hbox{$L$} \!\mathord{\left/ {\vphantom {L 2}}\right.\kern-\nulldelimiterspace} \!\lower0.7ex\hbox{$2$}},\, - \varepsilon_{0} } \right)$$ and $$\left( {{\raise0.7ex\hbox{$L$} \!\mathord{\left/ {\vphantom {L 2}}\right.\kern-\nulldelimiterspace} \!\lower0.7ex\hbox{$2$}},\,L + \varepsilon_{0} } \right)$$ for the wire locations give increase to the resulting system:

8 $$\frac{{\partial u_{3} }}{{\partial y_{3} }} = 0,$$

9 $$\frac{\partial p}{{\partial y_{1} }} = \frac{\partial p}{{\partial y_{2} }} = 0,$$

10 $$- \frac{\partial p}{{\partial y_{3} }} + \mu \left( {\frac{{\partial^{2} }}{{\partial y_{1}^{2} }} + \frac{{\partial^{2} }}{{\partial y_{2}^{2} }} + \frac{{\partial^{2} }}{{\partial y_{3}^{2} }}} \right)u_{3} + \left( {\rho \beta } \right)g\left( {T - T_{0} } \right) + \sigma \overline{\mu }_{0}^{2} \overline{H}^{2} u_{3} = 0,$$

11 $$u_{3} \frac{\partial T}{{\partial y_{3} }} = \alpha \left( {\frac{{\partial^{2} T}}{{\partial y_{1}^{2} }} + \frac{{\partial^{2} T}}{{\partial y_{2}^{2} }} + \frac{{\partial^{2} T}}{{\partial y_{3}^{2} }}} \right),$$

where $$\overline{H}^{2} = \left( {\frac{\gamma }{2\pi }} \right)^{2} \left( {\frac{1}{{\sqrt {\left( {y_{1} - {\raise0.7ex\hbox{$L$} \!\mathord{\left/ {\vphantom {L 2}}\right.\kern-\nulldelimiterspace} \!\lower0.7ex\hbox{$2$}}} \right)^{2} + \left( {y_{2} + \varepsilon_{0} } \right)^{2} } }} + \frac{1}{{\sqrt {\left( {y_{1} - {\raise0.7ex\hbox{$L$} \!\mathord{\left/ {\vphantom {L 2}}\right.\kern-\nulldelimiterspace} \!\lower0.7ex\hbox{$2$}}} \right)^{2} + \left( {y_{2} - L - \varepsilon_{0} } \right)^{2} } }}} \right)^{2} .$$

It is observable that the pressure is the function of y_3_ merely. It is well known (please see, Jha and Gambo^61^) that for the fully developed flow when the velocity distribution over any cross section of the duct does not change along the direction of flow (axial direction), the pressure gradient is a constant. It is observable that the pressure is the function of $y_{3}$ merely. Additional, for the completely established (hydrodynamically as well as thermally) movement under the condition of axially uniform heats fluxes and the constant wall temperature, it is recognized that $\frac{\partial p}{{\partial y_{3} }}{\text{and}}\frac{\partial T}{{\partial y_{3} }}\,$ is a fixed with:

12 $$\frac{\partial T}{{\partial y_{3} }} = \frac{{dT_{w} }}{{dy_{3} }} = \frac{{\partial T_{0} }}{{\partial y_{3} }} = 4\frac{{\dot{Q}}}{k\,L}\,,$$

where

$$\dot{Q} = \frac{k}{4L}\left( {\int\limits_{0}^{L} {\left( {\left. {\frac{\partial T}{{\partial y_{1} }}} \right|_{L} - \left. {\frac{\partial T}{{\partial y_{1} }}} \right|_{0} } \right)} dy_{2} + \int\limits_{0}^{L} {\left( {\left. {\frac{\partial T}{{\partial y_{2} }}} \right|_{L} - \left. {\frac{\partial T}{{\partial y_{2} }}} \right|_{0} } \right)} dy_{1} } \right)$$

is the fixed number incidentally be around wall heat flux.

An evident significance of Eq. (12) is the decrease of Eq. (11) to:

13 $$u_{3} \left( {4\frac{{\dot{Q}}}{kL}\,} \right) = \alpha \left( {\frac{{\partial^{2} T}}{{\partial y_{1}^{2} }} + \frac{{\partial^{2} T}}{{\partial y_{2}^{2} }}} \right).$$

Now, the succeeding dimensionless variables:

14 $$x_{1} = \frac{{y_{1} }}{L},\,x_{2} = \frac{{y_{2} }}{L},\,\,\theta = \frac{{T - T_{w} }}{{\left( {{\raise0.7ex\hbox{${\dot{Q}}$} \!\mathord{\left/ {\vphantom {{\dot{Q}} {kw_{m} }}}\right.\kern-\nulldelimiterspace} \!\lower0.7ex\hbox{${kw_{m} }$}}} \right)}},\,w = - \frac{{\mu u_{3} }}{{L^{2} \left( {{\raise0.7ex\hbox{${dp}$} \!\mathord{\left/ {\vphantom {{dp} {dy_{3} }}}\right.\kern-\nulldelimiterspace} \!\lower0.7ex\hbox{${dy_{3} }$}}} \right)}},\,H = \frac{{\overline{H}}}{{\overline{H}_{0} }},\,\varepsilon = \frac{{\varepsilon_{0} }}{L},$$

decrease the Eqs. (10) and (13) to:

15 $$1 + \left( {\frac{{\partial^{2} w}}{{\partial x_{1}^{2} }} + \frac{{\partial^{2} w}}{{\partial x_{2}^{2} }}} \right) + Ra\,\theta - M^{2} \,H^{2} w = 0,$$

16 $$\left( {\frac{{\partial^{2} }}{{\partial x_{1}^{2} }} + \frac{{\partial^{2} }}{{\partial x_{2}^{2} }}} \right)\theta = w.$$

where $\overline{H}_{0} = \frac{\gamma }{2\pi \varepsilon }$ is the maximum MF intensity at the channel shallow, utilized to familiarize the dimensionless MF strength H. Additional, $\varepsilon = \frac{{\varepsilon_{0} }}{L}$ is the conforming position of the dipole in the non-dimensional $x_{1}^{{}} x_{2}^{{}}$-coordinate system, and $\theta$ is the dimensionless temperature. In the present study, we have fixed $\varepsilon_{0} = \frac{L}{2}$ which consequently means that $\varepsilon = 0.5.$

## Numerical methodology

**Figure 2:**
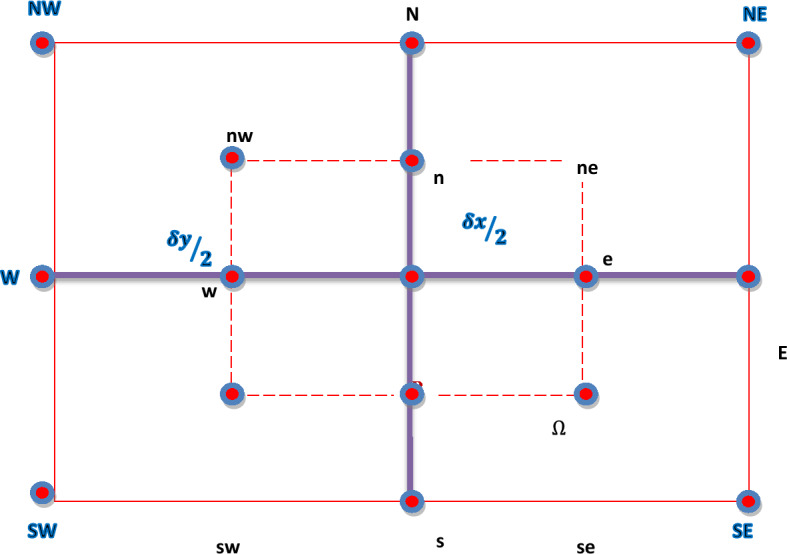
Control Volume around a General Grid Point P.

The partial differential equations (in algebraic form) are evaluated by means of finite volume method (FVM). The differential equations, in the FVM, are transformed to surface integrals and then solved iteratively. The system of algebraic partial differential equations can be solved with the usual numerical methods. But the unknown conditions such as e.g. initial or boundary conditions cause a trouble in finding the numerical solution. At some stage, the system might be divergence even for precise estimations of missing conditions. Contrarily, solution will be interrupted for the partial differential equations involving the complex eigen values. However, finite volume method is the best choice to tackle such types of problems which might not be fixed easily by the other methods. On the other hand, a better convergence can be obtained with FVM as compared to other numerical methods. Obviously, Eqs. (15 and 16) may be put in the general form:

17 $$\frac{{\partial^{2} f}}{{\partial x_{1}^{2} }} + \frac{{\partial^{2} f}}{{\partial x_{2}^{2} }} = g(x_{1} ,x_{2} ),$$

with $f(x_{1} ,x_{2} )\,{\text{and}}\,g(x_{1} ,x_{2} )$ being the unknown and the known functions (respectively). For the finite volume discretization (on the regular structured mesh) of Eq. (17), the general point $P\left( {x_{1} ,\,x_{2} } \right)$ is assumed to be surrounded by the points *E, N, S, W* etc. (as shown in the Fig. 2 below). For discretization purpose, Eq. (17) is first integrated over the control volume, shown in Fig. 2, and further simplifications are performed as follows:

18 $$\mathop \smallint \limits_{{x_{1w} }}^{{x_{1e} }} \mathop \smallint \limits_{{x_{2s} }}^{{x_{2n} }} \left( {\frac{{\partial^{2} f}}{{\partial x_{1}^{2} }} + \frac{{\partial^{2} f}}{{\partial x_{2}^{2} }}} \right)dx_{2} dx_{1} = \mathop \smallint \limits_{{x_{1w} }}^{{x_{1e} }} \mathop \smallint \limits_{{x_{2s} }}^{{x_{2n} }} g(x_{1} ,x_{2} )dx_{2} dx_{1}$$

It is to point out that the control volume is defined by $x_{1w} \le x_{1} \le x_{1e} ,x_{2s} \le x_{2} \le x_{2n}$.

Now we integrate and evaluate the integrals over each term in Eq. (18) as given below:

19 $$\begin{aligned} \mathop \smallint \limits_{{x_{1w} }}^{{x_{1e} }} \mathop \smallint \limits_{{x_{2s} }}^{{x_{2n} }} \frac{{\partial^{2} f}}{{\partial x_{1}^{2} }}dx_{2} dx_{1} & = \mathop \smallint \limits_{{x_{2s} }}^{{x_{2n} }} \left[ {\left( {\frac{\partial f}{{\partial x_{1} }}} \right)_{{x_{1e} }} - \left( {\frac{\partial f}{{\partial x_{1} }}} \right)_{{x_{1w} }} } \right]dx_{2} \\ & = \mathop \smallint \limits_{{x_{2s} }}^{{x_{2n} }} \left( {\frac{{f_{E} - f_{P} }}{{\delta x_{1} }}} \right)dx_{2} - \mathop \smallint \limits_{{x_{2s} }}^{{x_{2n} }} \left( {\frac{{f_{P} - f_{W} }}{{\delta x_{1} }}} \right)dx_{2} = \left( {\frac{{f_{E} - f_{P} }}{{\delta x_{1} }}} \right)\left( {x_{2n} - x_{2s} } \right) - \left( {\frac{{f_{P} - f_{W} }}{{\delta x_{1} }}} \right)\left( {x_{2n} - x_{2s} } \right) \\ & = \frac{{\delta x_{2} }}{{\delta x_{1} }}\left( {f_{E} - 2f_{P} + f_{W} } \right) \\ \end{aligned}$$

Similarly, the integration over the second term leads to:

20 $$\mathop \smallint \limits_{{x_{1w} }}^{{x_{1e} }} \mathop \smallint \limits_{{x_{2s} }}^{{x_{2n} }} \frac{{\partial^{2} f}}{{\partial x_{2}^{2} }}dx_{2} dx_{1} = \frac{{\delta x_{1} }}{{\delta x_{2} }}\left( {f_{N} - 2f_{P} + f_{S} } \right)$$

Finally, incorporation of Eqs. (19–20) in Eq. (18) yields:

21 $$\frac{{\delta x_{2} }}{{\delta x_{1} }}\left( {f_{E} + f_{W} } \right) + \frac{{\delta x_{1} }}{{\delta x_{2} }}\left( {f_{N} + f_{S} } \right) - 2\left( {\frac{{\delta x_{1} }}{{\delta x_{2} }} + \frac{{\delta x_{2} }}{{\delta x_{1} }}} \right)f_{P} \delta x_{1} \delta x_{2} = g_{P} \delta x_{1} \delta x_{2}$$

**Figure 3:**
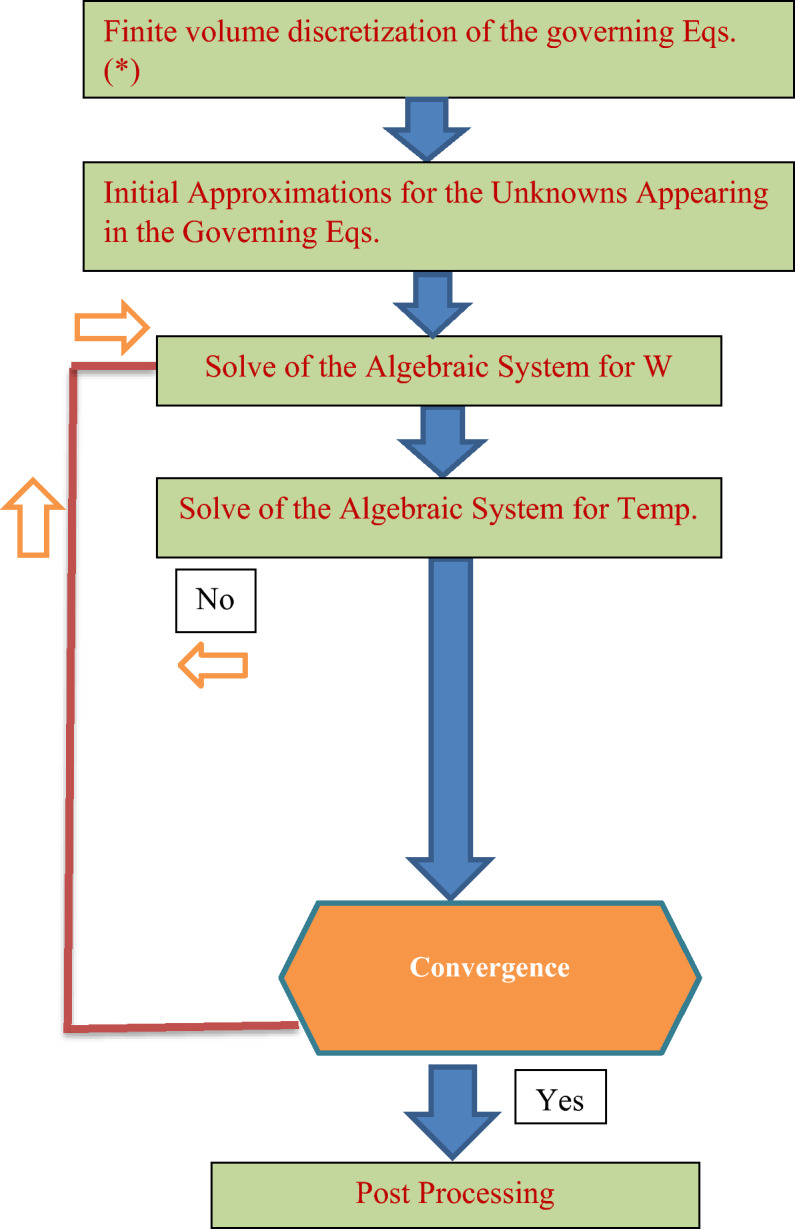
Flow Chart for the Numerical Solution.

The algebraic system, in light of Eq. (21), is corresponding to the overriding Eqs. (15–16) is lastly resolved iteratively. The procedure steps for the present technique may be shown as in Fig. 3.

## Results and discussions

**Figure 4:**
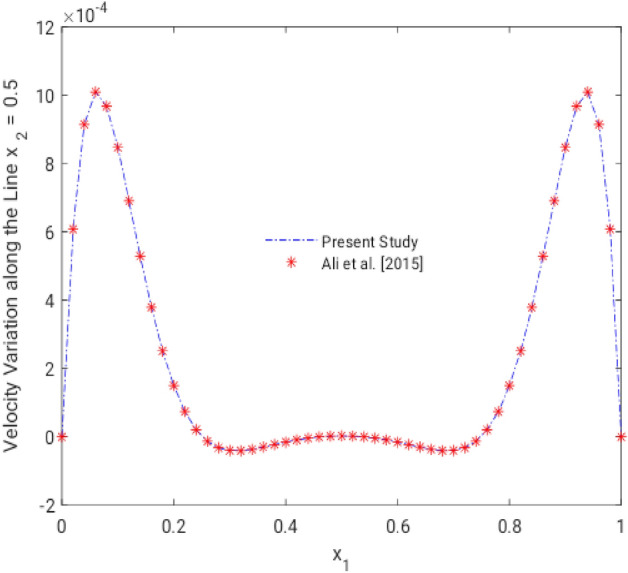
Comparison of present numerical results with scientific literature^8^.

Our numerical results for the flow velocity in the central of the channel along the straight line, for the case when there is no wire, compare favorably with the existing literature (Ali et al.^8^), as shown in the Fig. 4.

Results of the parametrical studies on flow and thermal dispersal through the rectangular duct which contains current carrying wires were discussed in detail. Crucial parametric constrains were considered to be the Rayleigh Number and the MF which was induced by the current carrying wires in the duct. Figures 5, 6, 7, 8, 9, 10, 11, 12, 13, 14, 15, 16, 17, 18, 19, 20, 21, 22, 23, 24, 25 and 26 depicts the behaviour of the flow fluid and the thermal distribution in the duct with three and two dimensional plots.

**Figure 5:**
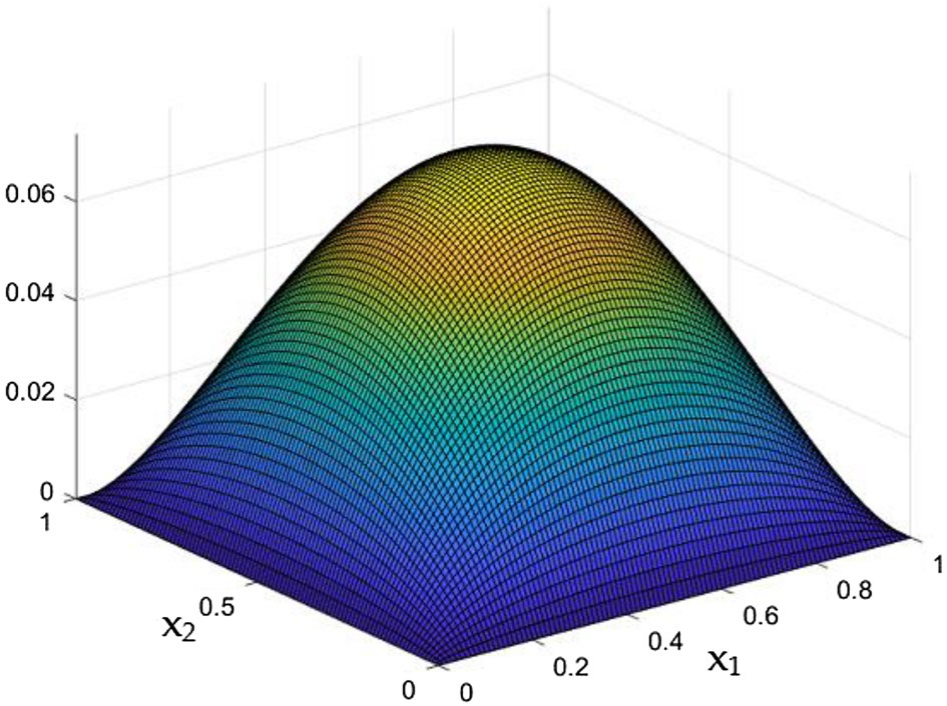
Velocity field for Ra = 0, in the absence of external MF.

Figure 5 exposes the un interrupted flow nature in the duct in the absence of MF and Rayleigh Number. It clearly shows the smoother flow which were slower near the wall and at the core it moves faster. The product of the Prandtl number (Pr) and the Grashof number (Gr) can be referred to as the Rayleigh number e. g. Ra = Gr × Pr. It is worth mentioning here that the correlation of viscosity and buoyancy within a fluid is described by the Grashof number. Whereas the Prandtl number expresses the relationship between thermal diffusivity and momentum diffusivity. However, Rayleigh number characterizes the heat transport in the phenomenon of natural convection. Heat transfers due to thermal conduction below the critical value of Rayleigh number (Ra = 1708).

**Figure 6:**
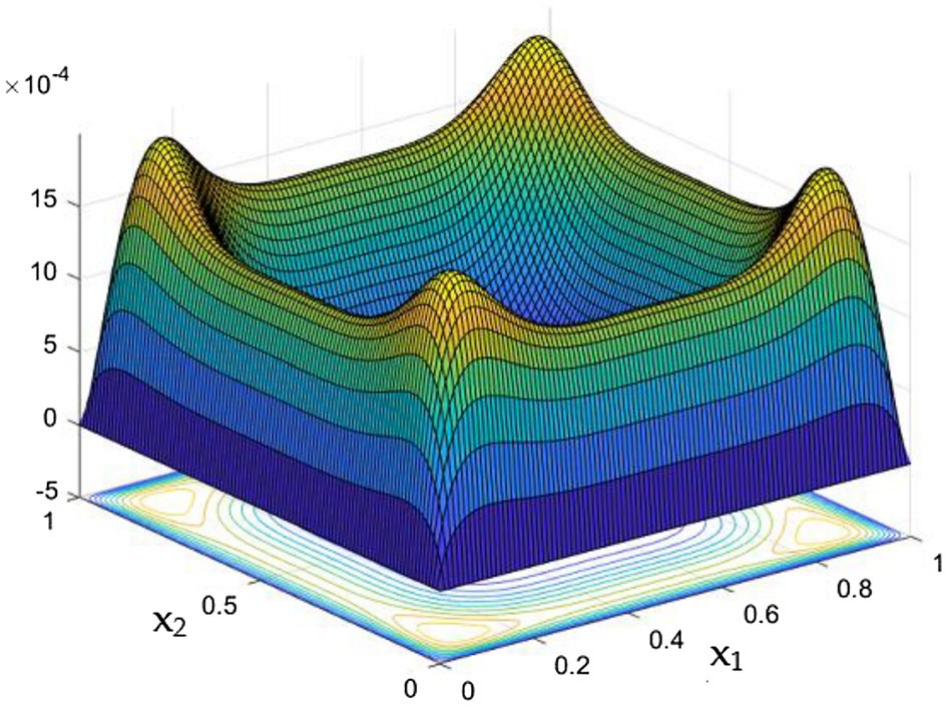
Velocity field for Ra = 50,000, in the absence of external MF.

**Figure 7:**
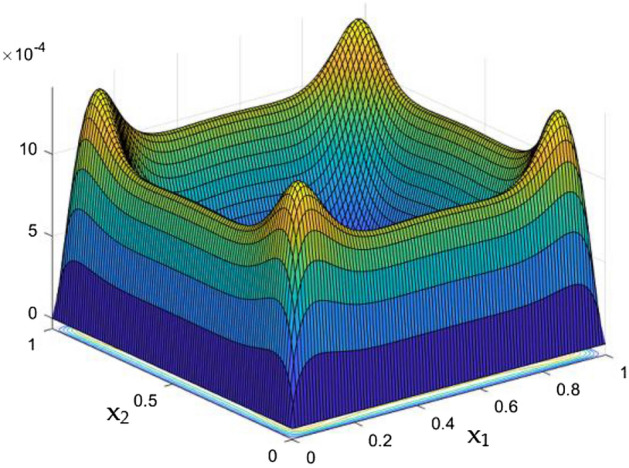
Velocity field for Ra = 100,000, in the absence of external MF.

**Figure 8:**
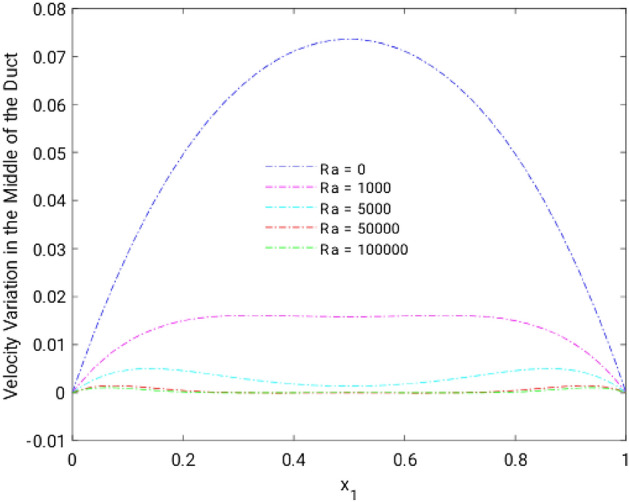
Velocity variation along the line $x_{2} = 0.5$ for altered Ra, in the absence of external MF.

Figures 6, 7 and 8 showcased the exclusive impact of Rayleigh Number (Ra) over the flow in the duct in without any influence of MFs. It can be clearly noted the increase velocity changes in the corners than in the core region for the Rayleigh number increase. This may due to the fact that, the flow nature alterations induced by the Rayleigh number improvement were more significant in the top corners and the wall than the core region of the duct. In the core of the duct, Rayleigh number impact were getting dominated by the flow which was not interrupted by the MFs.

**Figure 9:**
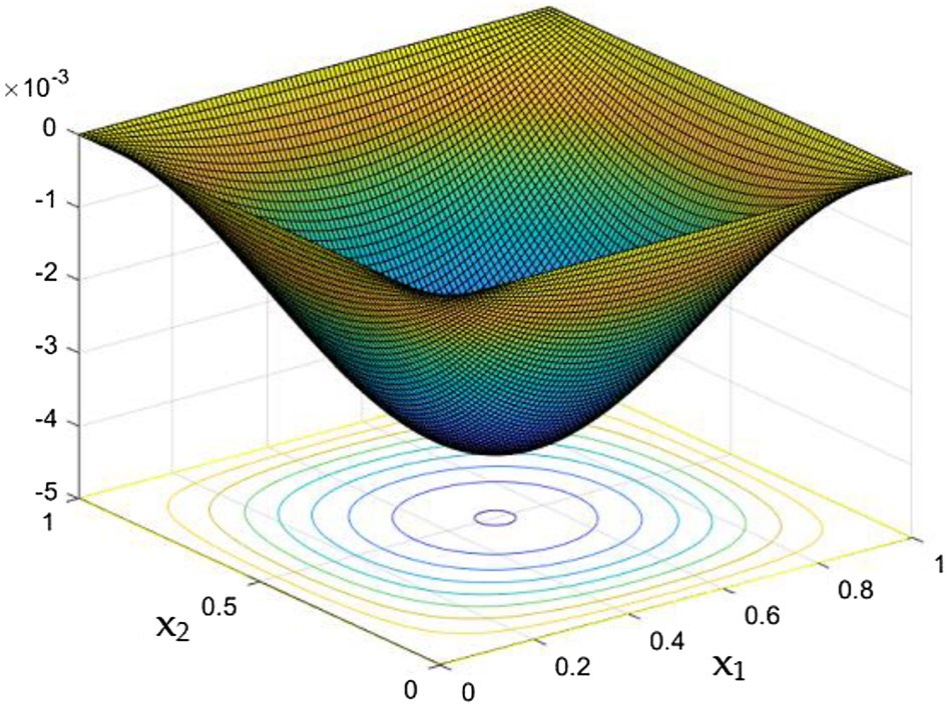
Temperature field for Ra = 0, in the absence of external MF.

Unaltered thermal dispersal in the rectangular duct were portrayed in the Fig. 9 in absence of Rayleigh number and the MFs. As the thermal distribution in the duct gets correlated with the flow nature, it was higher in the leading edge of the duct and slowly decelerates towards its core. The intense flow in the core without any frictional loss from the wall were able to wipes the more temperature in the duct when compared to the situations near the wall and corners.

**Figure 10:**
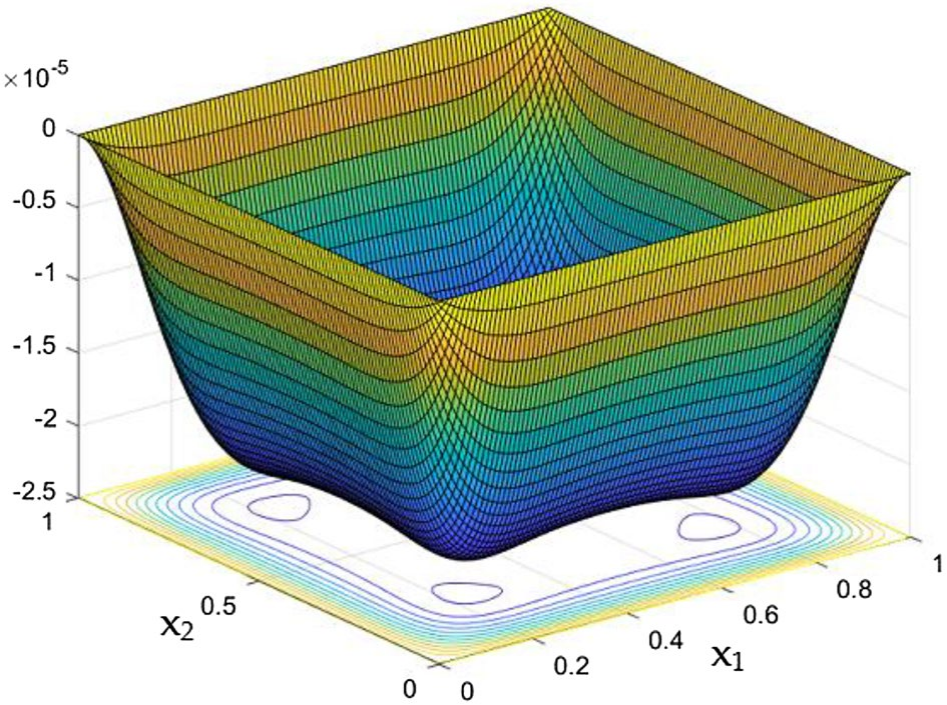
Temperature field for Ra = 50,000 in the absence of external MF.

**Figure 11:**
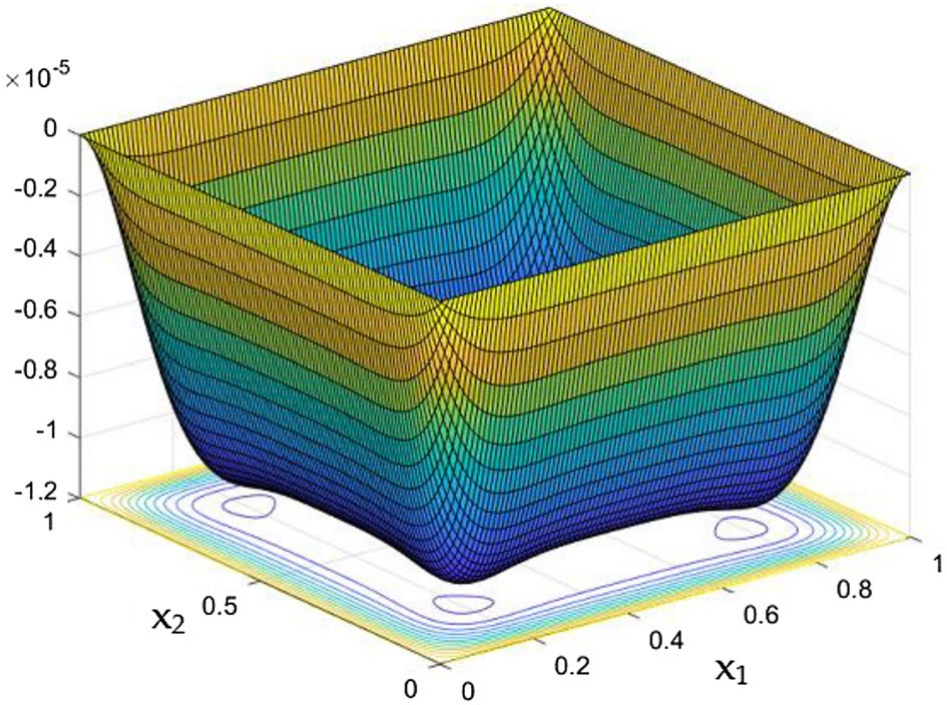
Temperature field for Ra = 10,000 in the absence of external MF.

**Figure 12:**
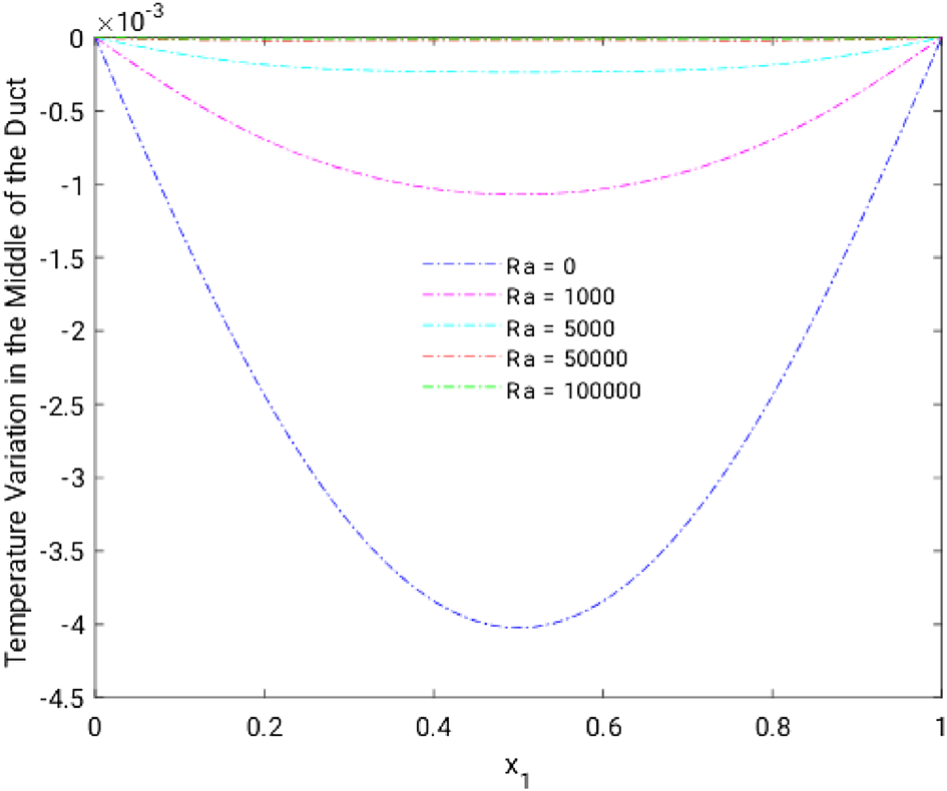
Temperature variation along the line $x_{2} = 0.5$ for different Ra, in the absence of external MF.

**Figure 13:**
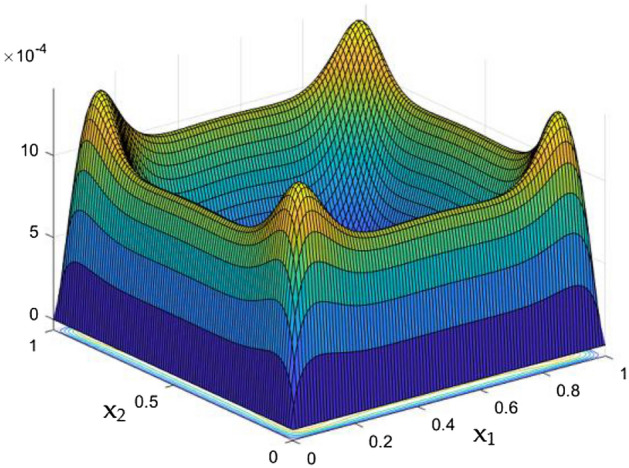
Velocity field for Ra = 100,000 and M = 0.

**Figure 14:**
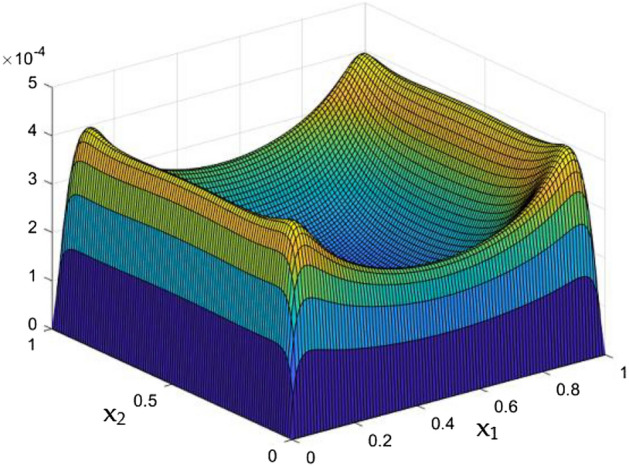
Velocity field for Ra = 100,000 and M = 20.

The thermal dispersion in the duct is highlighted by plots from Figs. 10, 11 and 12 for changes in the Rayleigh number without any effects from the MF. While the Rayleigh number increased the thermal dispersal ability of the flow gets disturbed due to flow nature alteration happening in it. This reflects in the plots which shows the higher thermal distributions in the leading phases of the duct as it goes deeper the dispersal getting reduced.

**Figure 15:**
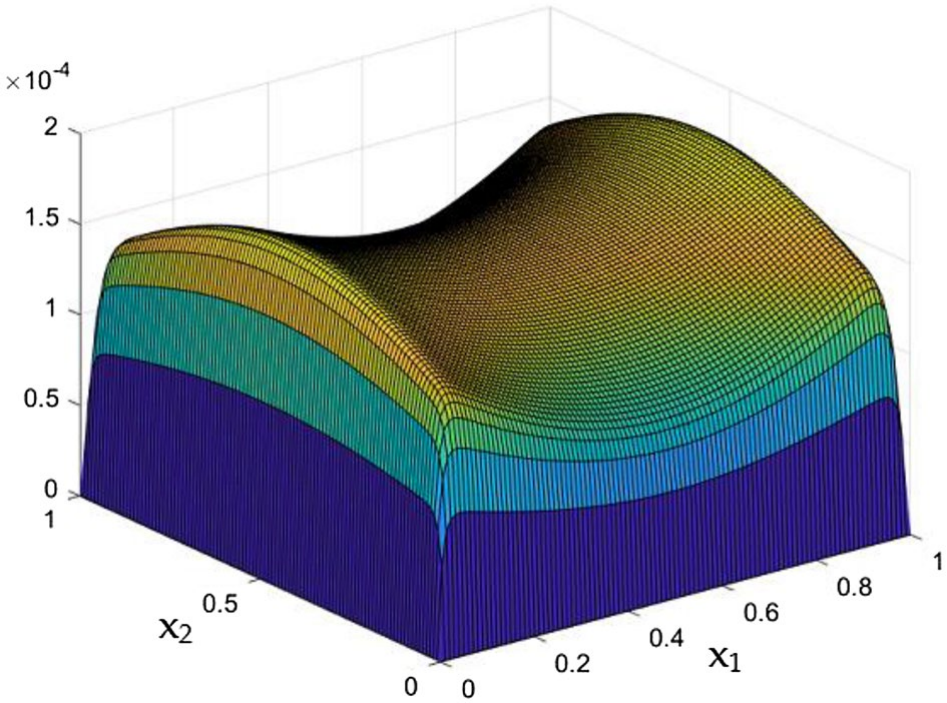
Velocity field for Ra = 100,000 and M = 40.

**Figure 16:**
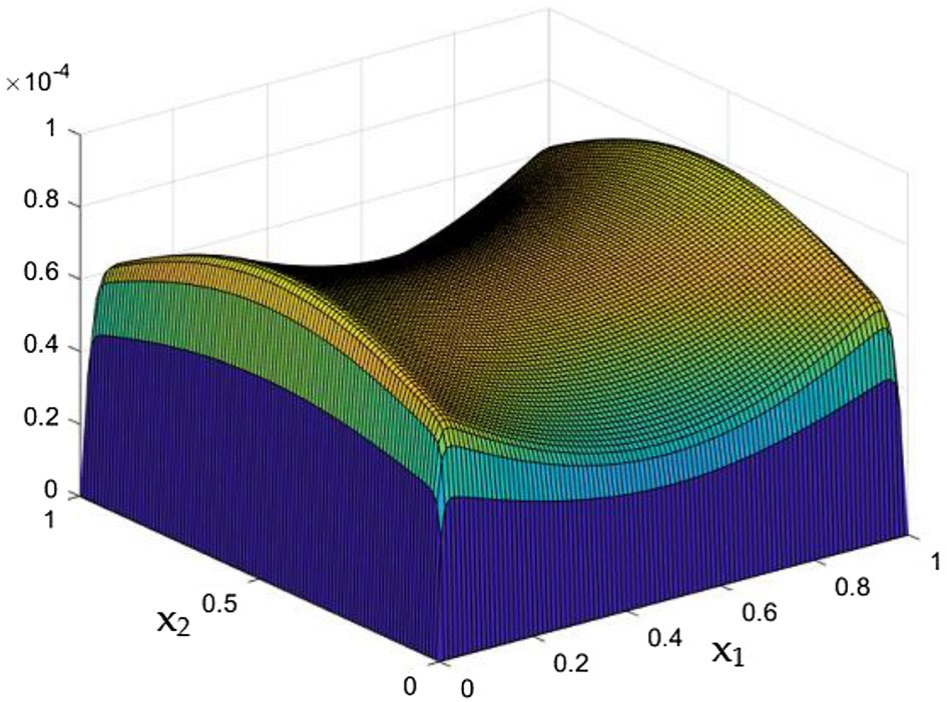
Velocity field for Ra = 100,000 and M = 60.

**Figure 17:**
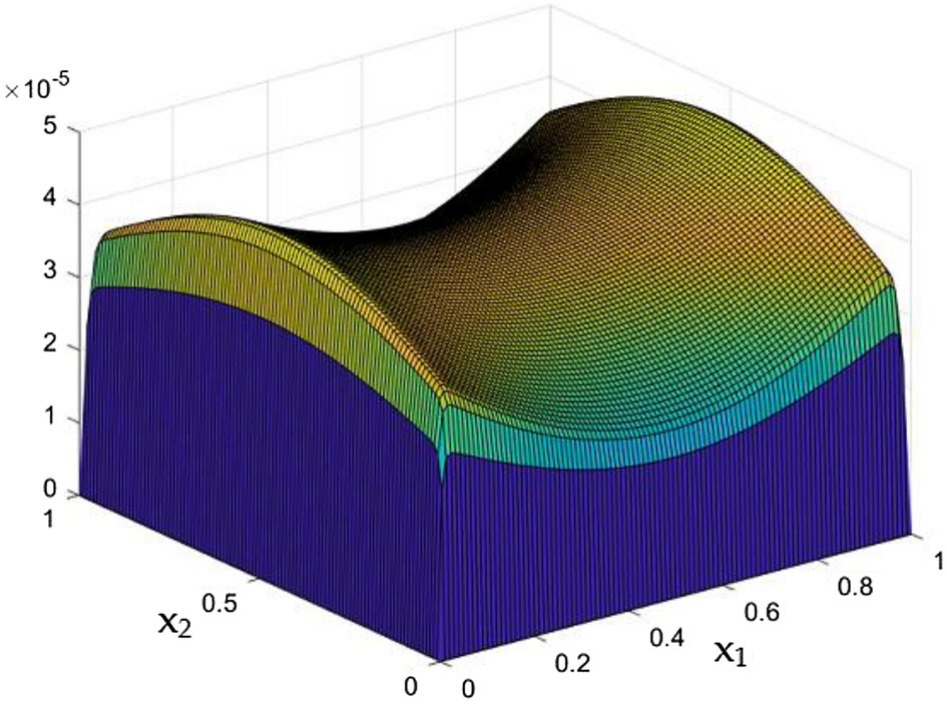
Velocity field for Ra = 100,000 and M = 80.

**Figure 18:**
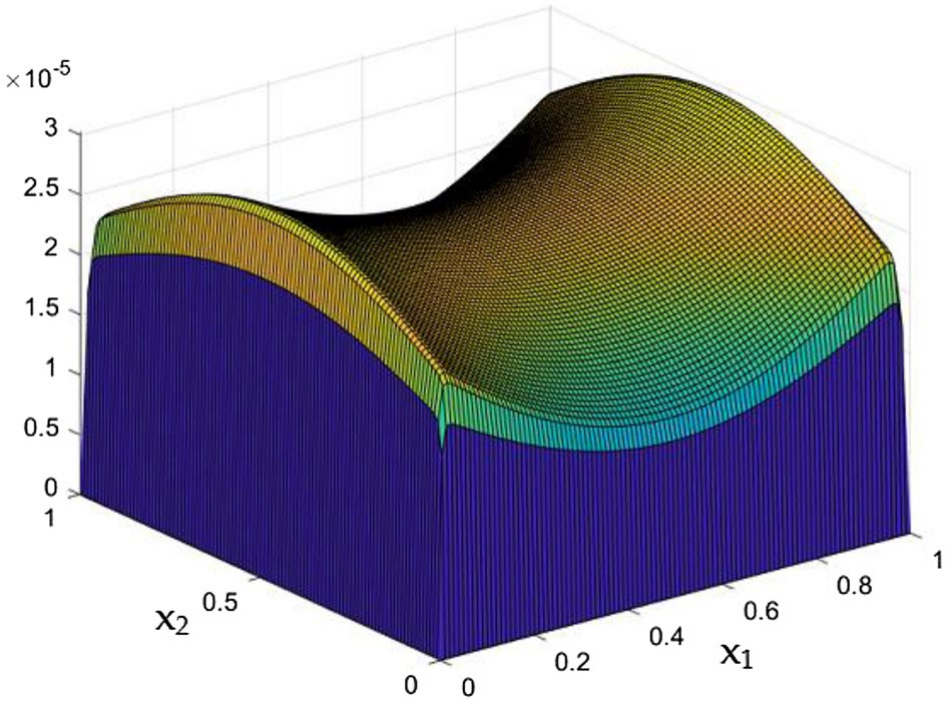
Velocity field for Ra = 100,000 and M = 100.

**Figure 19:**
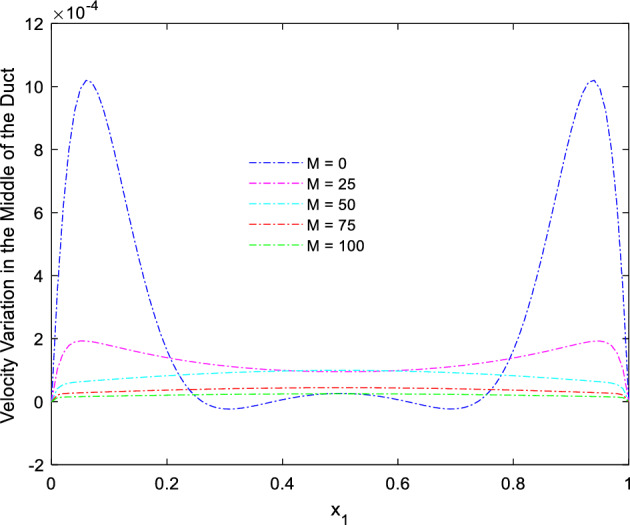
Velocity variation along the line $x_{2} = 0.5$ for Ra = 100,000 and different *M.*

While the current started to pass through the carrying wires in the rectangular duct, it induces MFs which can influence both the flow and thermal dispersal in the duct. Figure arrays from Figs. 13, 14, 15, 16, 17, 18 and 19 demonstrates the velocity changes occurs in the duct for improving MF strength at a consistent Rayleigh number around Ra = 100,000.

**Figure 20:**
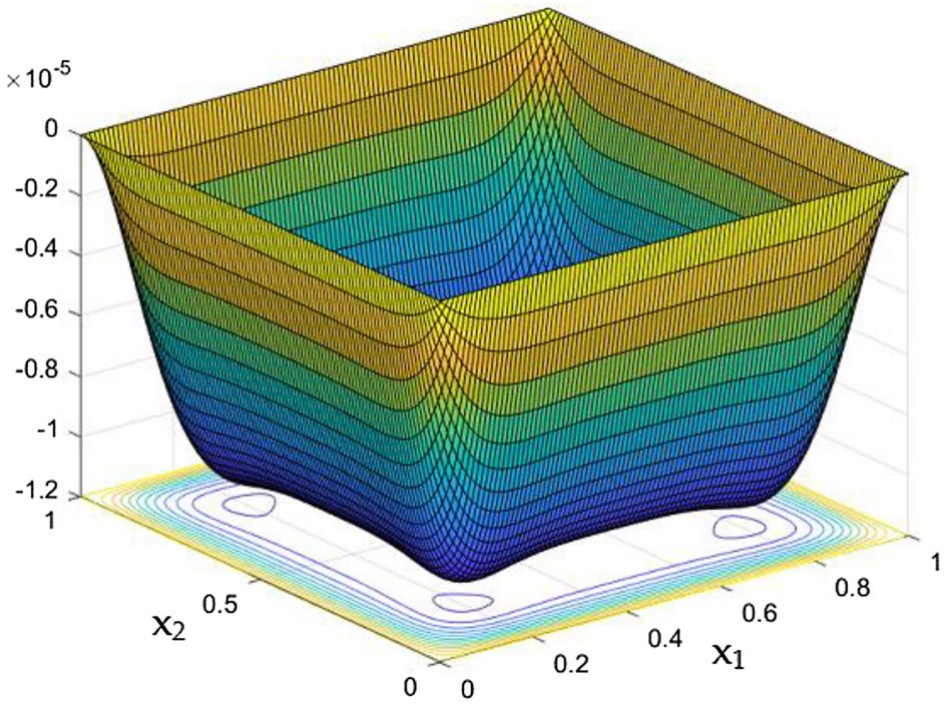
Temperature field for Ra = 100,000 and M = 0.

**Figure 21:**
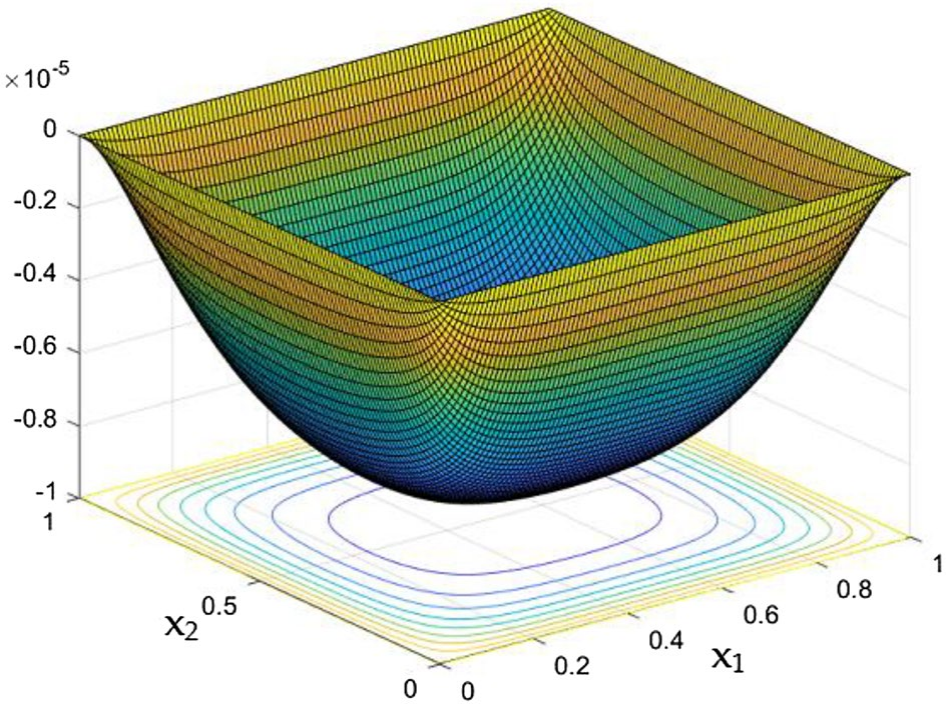
Temperature field for Ra = 100,000 and M = 20.

**Figure 22:**
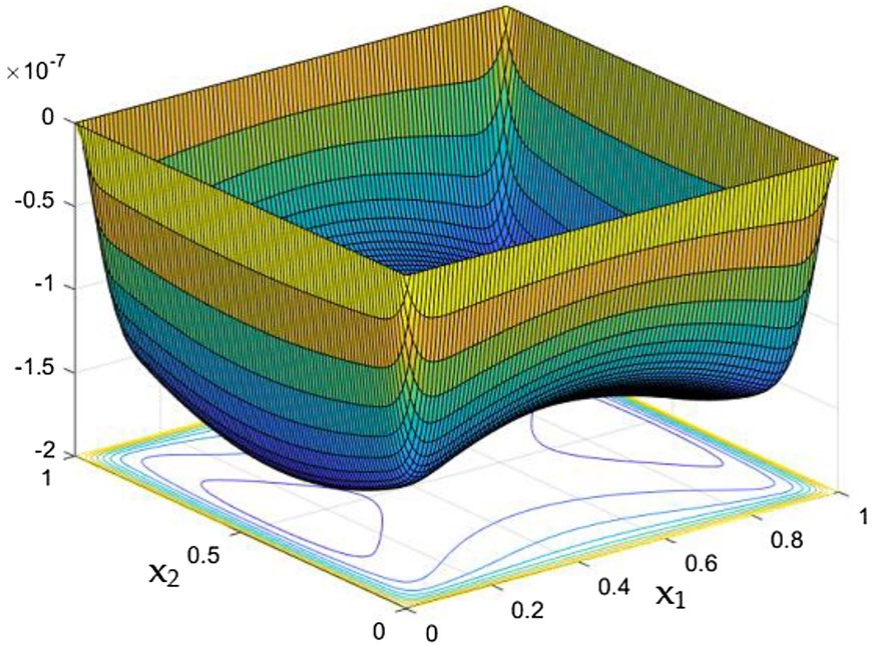
Temperature field for Ra = 100,000 and M = 40.

**Figure 23:**
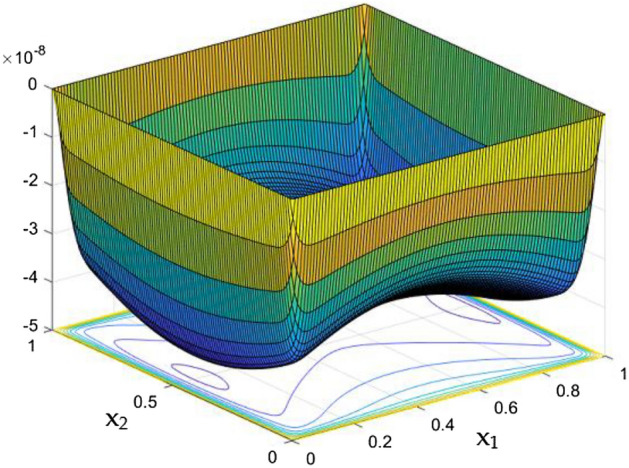
Temperature field for Ra = 100,000 and M = 60.

This consideration of Rayleigh number ensures to excludes the flow nature alteration and provides improved results towards the MF impacts. Initially in the absence of MF the flow looks intense in the corners irrespective of the sides of the walls. Once the current started to pass and the MFs emerges notable changes can be appeared in the flow field. For the initial values of the MF strength the flow near the leading edges of the wall closer to the current wire looks dominant than the core and dent in the core flow field was noted.

Interestingly, Figs. 15 and 16 evident the flow pattern change occurs in the duct once after the MF gets more dominate and cover the duct area. Particularly, between M = 40 and M = 60 the dent gets disappeared and the tomb shape started to develop like connecting the wall holding the wires. This may due to the squeezing happening in the either wall which was not holding the current wires where the MF was not dominative. From there the trend sustains for further values of higher magnetic strength which can be detected as of Figs. 17 and 18.

**Figure 24:**
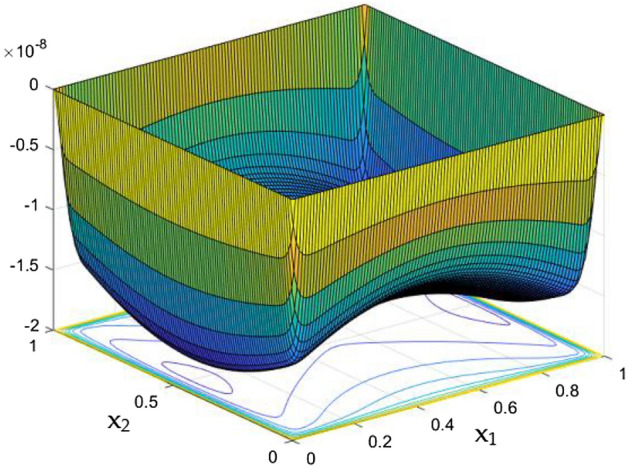
Temperature field for Ra = 100,000 and M = 80.

**Figure 25:**
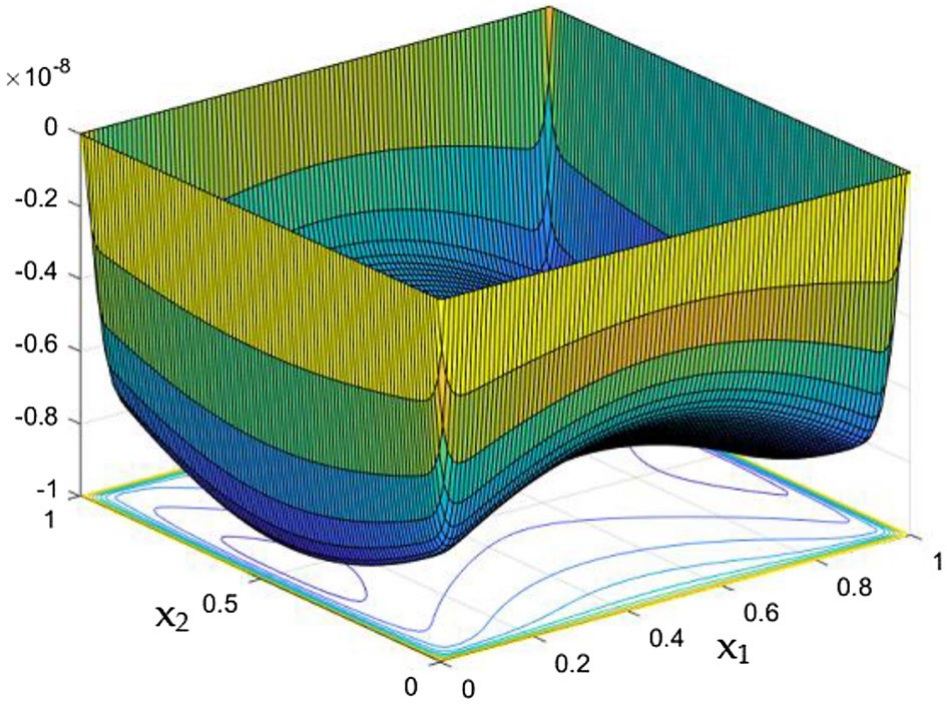
Temperature field for Ra = 100,000 and M = 100.

**Figure 26:**
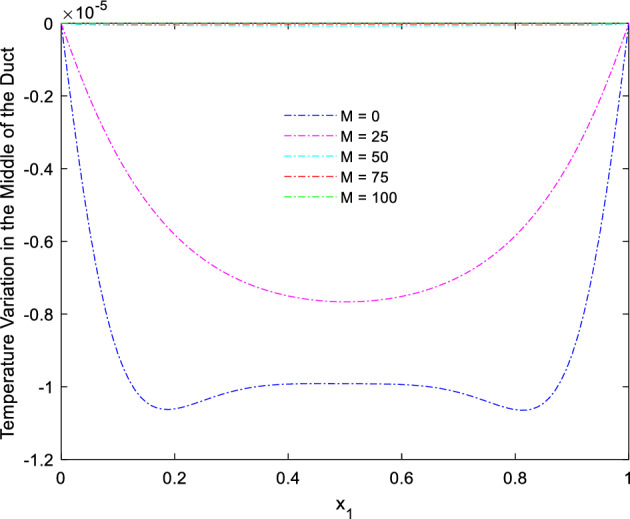
Temperature variation along the line $x_{2} = 0.5$ for Ra = 100,000 and different *M.*

The collective plots from Figs. 20, 21, 22, 23, 24, 25 and 26 elucidate the temperature field variations with respect to the MF development around the duct. It is clearly observed from the gaps develops at the bottom of the plots that, the temperature in the further end of the duct noticeably nominal. At the initial stages of MF, the thermal dispersal occurs evenly around the wall and lower towards the duct core. As like in the flow field, in between the crucial range of M = 40 and M = 60 the temperature field also underwent the significant changes. Corresponding to the velocity squeezing in the other ends of wired sides, the thermal field experiences the substantial difference the temperature dispersion between the walls. As the MF strengthens, the wired holding side possess deeper thermal traces while comparing to the other two sides. This may due to the fact that, the swifter velocity in the further side drives the temperature faster than the wire holding side with reduced velocity. Figure 24 discloses the two dimensional view of the above mentioned MF behaviors over the temperature distribution over the rectangular duct. Higher the magnetic strength, the temperature traces end closer to the leading edge of the duct itself.

## Conclusions

Impact of the two nearby current carrying wires on the momentum and temperature behavior in the flow (driven by external pressure gradient) inside a vertical duct has been numerically investigated. In order to validate our computational technique, the numerical results have been compared, and are found to be in excellent comparison with the ones reported in existing literature. Based on the numerical study, following conclusions may be drawn:

- Rayleigh number holds a significant influence over the velocity field in the rectangular duct irrespective of location of the wires.
- Compared to the region near the walls, Rayleigh number is more influential for the flow in the duct center.
- The MF caused by the wires has been found to act against the flow reversal (at high Raleigh number). In this way the MF tends to balance the impact of buoyancy in the laminar flow regime.
- Thermal distribution is significantly reduced over the whole duct, as the MF is strengthened.
- It may be inferred that the flow reversal may be controlled by applying a MF of appropriate power, around the channel, carrying the flow.

### Future direction

Future extensions of the present study include but not limited to:

- Various numerical experiments may be performed with different types of fluids of industrial interest. For example, Nanofluids, Hybrid nanofluids, Casson fluids etc.
- Rectangular duct can be replaced by the other shapes of ducts (e.g., circular, elliptical or wavy etc.)
- Entropy changes may also be studied with wide range of combinations in fluid choices and duct shapes.
- The Finite volume method could be applied to a variety of physical and technical challenges in the future^62–78^.

## Data Availability

All data generated or analyzed during this study are included in this published article.
